# Modeling Infant *i*'s Look on Trial *t*: Race-Face Preference Depends on *i*'s Looking Style

**DOI:** 10.3389/fpsyg.2017.01016

**Published:** 2017-06-23

**Authors:** Hoben Thomas, Ina Fassbender

**Affiliations:** ^1^Department of Psychology, Pennsylvania State UniversityUniversity Park, PA, United States; ^2^Department of Developmental Psychology, Ruhr-University BochumBochum, Germany

**Keywords:** infant looking, face preference, mixture models, looking styles

## Abstract

When employing *between-infant* designs young infants' looking style is related to their development: Short looking (SL) infants are cognitively accelerated over their long looking (LL) peers. In fact, looking style is a *within-infant* variable, and depends on infant *i*'s look distribution over trials. For the paired array setting, a model is provided which specifies the probability, π_*i*_ ∈ [0, 1], that *i* is SL. The model is employed in a face preference study; 74 Caucasian infants were longitudinally assessed at 3, 6, and 9 months. Each *i* viewed same race (Caucasian) vs. other race (African) faces. Infants become SL with development, but there are huge individual differences in rate of change over age. Three month LL infants, ${\hat{\pi }}_{i}<1/2$, preferred other race faces. SL infants, ${\hat{\pi }}_{i}>1/2$, preferring same race faces at 3, and other race faces at 6 and 9 months. Looking style changes precede and may control changes in face preference. Ignoring looking style can be misleading: Without considering looking style, 3 month infants show no face preference.

## 1. Introduction

Colombo et al. have shown that an infant's looking style which refers to an infant's duration of visual fixations to a stimulus, is an important indicator of cognitive development. Infants with short visual fixations (short looking, or SL) are cognitively advantaged over infants with longer visual fixations (long looking, or LL) (Colombo et al., 2011). However, gauging the importance of looking style has been sharply constrained for two reasons: First, looking style has only been assessed at the *between-infant* level, with infants classified LL or SL. In fact, looking style is a *within-infant* phenomenon. Second, to assess looking style has required a pretest. So the potential importance of looking style can only be assessed in settings which employ the pretest, which additionally, adds “overhead” to any setting, making subject loss more probable. These difficulties are eschewed in the model detailed here which assesses looking style at the within-infant individual-look level and without additional technology. The model could potentially unlock unrecognized information on video tapes long relegated to storage, if data were collected under the paired stimulus preference paradigm, pioneered by Fantz (1958, 1961), the setting of focus here. Thus, infant fixations show dual use: First, for assessing an infant's short or long looks on each trial, and second for addressing whatever the primary goal of the study might be. The requirements for the model implementation are a sufficient number within-infant trial replications, an issue considered later. Importantly, the model and associated procedure allows for the assessment of how looking style impacts the major variable of focus.

Historically, Beasley (1933) was probably the first researcher to recognize that individual infants displayed a variety of different looking behaviors to various visual stimulus objects. But he did not know the cognitive implications of his observations. SL infants are cognitively advanced over their LL peers on a variety of cognitive tasks (see Colombo et al., 2011 for a review). Many researchers have observed infant looking differences although the terms used to characterize the differences have varied widely (Beasley, 1933; Stechler and Latz, 1966; Cohen, 1972; Haith, 1980; Bronson, 1982, 1994; Colombo et al., 1988; Hood, 1995; Butcher et al., 2000).

The pretest procedure was developed by Colombo and his colleagues in the late 1980s and can be viewed as a two-stage procedure. First it involves partitioning infants into two groups, SL and LL based on a median split of infants' “peak” pretest looking times (Colombo et al., 1988, 1995). Various procedures may follow in the second stage. Subsequently, the pretest is used to inform the primary analysis, with the pretest acting effectively as a covariate. The general paradigm continues to be employed (Courage et al., 2004; Diaz and Bell, 2011; Cuevas and Bell, 2014).

The pretest procedure can be viewed as a simple empirical decision rule without a decision-theoretical conceptual foundation. If forces half of any sample to be classified as SL infants, an implausible outcome given different infants, tasks, and settings. The procedure could be modified so it has a decision-theoretical probabilistic basis; however, this goal is not considered here.

The larger implication may be that looking style and particularly SL is important in a much wider range of developmental processes not currently identified as being impacted by looking style differences. To investigate these possible implications means an infant's looks must be able to be assessed within the context of the ongoing study, at the within-infant trial level. Aslin has remarked that “…all looking times include a mix of active information processing and blank stares (Aslin, 2007, p. 50).” The proposed model framework illustrates how within-infant looking style can be assessed. The theory allows each looking trial response to be probabilistically specified as whether it is long or short, for each *i* and for each trial *t*.

The methodology uses mixture modeling (e.g., Everitt and Hand, 1981; Titterington et al., 1985; McLachlan and Peel, 2000) detailed in the Appendix (Supplementary Materials) but at the within-subject level, a rarity. Thus, each *i* has her own model and associated parameters. The key is to view the looking style problem as a latent variables problem. What is latent, that is, unobserved or unknown, is whether an infant's look on trial *t* is long or short.

This article has two goals: First, it illustrates how within-infant trial level inference is possible. Second, it illustrates how the information so culled can inform substantive research questions. Here the focus is on the development of preferences for faces of one's own race (also referred to as same race) vs. preference for faces of some other race. Infants were longitudinally assessed at 3, 6, and 9 months. The methodology and infant ages are similar to some other studies (e.g., Liu et al., 2015) but employs longitudinal data. Consequently, a richer set of questions can be addressed.

Briefly, the research concerning same race and other race face preferences is related to the larger literature on other race effects, but a preference paradigm is employed. Among newborns, neither same race nor other race faces have been preferred, but preference for own-race faces has been demonstrated by 3 month infants from different ethnic groups (Kelly et al., 2005, 2007; Bar-Haim et al., 2006; Liu et al., 2015). Other race face preference has been demonstrated at 9 months, but not at 6 months (Liu et al., 2015).

Among the issues considered here are the following: (1) Infants are provided with their own probability parameter π_*io*_ the probability of infant *i* being a short looker at age *o* (in what follows, *o* is often suppressed). So the change in *i*'s looking style may be more precisely indexed than previously possible. (2) What role does looking style play in the development of same or other race face preference? (3) How are looking style changes and race-face preferences intertwined in development? Do looking style changes precede, follow, or develop concurrently with changes in race face preferences? (4) A longitudinal approach allows for better understanding of an individual infant's face preference over age which is reflected in several analyses.

## 2. Methodology

### 2.1. Historical motivation

Suppose two infants each have four looking trials for some task. Let the four trial response looking times be for one infant 1, 1, 1, 1 and for the other 4, 0, 0, 0. Cohen (1972) argued that although two infants could have the same total (or average) looking times this does not tell the whole story, and “may obscure other information” (p. 870). Viewed as looking style differences in today's language, the first infant would be regarded as SL, the second LL. Cohen observed “It is difficult to believe that the same underlying attentional mechanism can account for both types of behavior” (p. 870). Cohen's concern has long been recognized (Colombo et al., 2011) but his concern has never been addressed at the level at which he implicitly expressed it: Using *within-infant* “other information” in Cohen's words to address *between-infant* similarities or differences. That is done here by considering the *distribution* of each infant's own looking trial responses. Different infant response distributions imply different infant behavior and different π_*i*_, where π_*i*_ is the probability *i* is a SL infant. Averaging over different infants' responses is still used to address substantive questions of focus, but these averages are conditional: i.e., the averages are conditioned on π_*i*_, *i* = 1, 2, 3, …, *n*, where *n* is sample size. Cohen refers to infants as “types,” a long-held assumption that implies looking style is a categorical variable. However many infants display responses of *both* types, as Aslin (2007) observed. Precisely, estimated π_*i*_ (denoted ${\hat{\pi }}_{i}$) range from from zero to one. So looking style, indexed by π_*i*_, is a continuous variable. The typology can be preserved by defining *i* as SL if π_*i*_ > 1/2 and as LL if π_*i*_ < 1/2.

Consider infant *i* 2523, at 3 months who viewed stimuli in a paired stimulus array (the setting will be described more completely below). *i*'s response on trial *t*, is the time viewing stimuli from set C (Caucasian faces) minus time spent viewing stimuli from set A (African faces) for *t* = 1, 2, …, *T* = 24 trials, an observed difference denoted *d*_*it*_ with −130 ≤ *d*_*it*_ ≤ 130 the result of counts of video frames. When *d*_*it*_ < 0 *i* is looking longer at an African face. When *d*_*it*_ > 0 *i* is looking longer at a Caucasian face. *i*'s vector of responses with *d*_*i*1_ = −117, *d*_*i*2_ = 120, …, *d*_*i*24_ = 0:

$$\begin{array}{cccccccccccc} -117 & 120 & -124 & 130 & 97 & 112 & -108 & 126 & -130 & -34 & -122 & 129\\ 129 & -76 & 103 & -103 & 0 & 0 & -117 & 130 & -112 & -108 & 115 & 0 \end{array}$$

Note the huge variability of the *d*_*it*_. One might suspect that *i* 2523 is displaying both long and short looks. When |*d*_*it*_| is 130, she spends all of her time on one stimulus. On trials for which |*d*_*it*_| is smaller such as 34, or 76, she distributes her looking times to each stimulus more equitably, and when *d*_*it*_ = 0 each is viewed for the same length of time. As will be seen, the model framework agrees with this intuition, *i*'s looking is “mixed.” But which looks are long and which short? Probabilistic answers can be given, and once answered her estimate ${\hat{\pi }}_{i}$ is immediately given. The average over trials $24^{-1}\underset{t}{\sum }d_{it}={\bar{d}}_{i}$ is *i*'s substantive summary variable, but the use of the ${\hat{\pi }}_{i}$ to inform the analysis of the ${\bar{d}}_{i}$ can be viewed as explicitly addressing Cohen's (1972) concern.

The basic approach, as noted, uses finite mixture theory and regards long and short responses for *i* to have distinct probability distributions. *i*'s long responses are assumed to follow one distribution. *i*'s short responses follow the other distribution. Thus, the estimation issue is specifying which distribution most probably gives rise to *d*_*it*_ for infant *i*'s trial *t*. Technical details, including estimation procedures and algorithms are given in the Appendix (Supplementary Materials).

### 2.2. General methodology

#### 2.2.1. Subjects

Seventy-four (35 female, 39 male) healthy full-term Caucasian infants were recruited for longitudinal assessment at 3, (90–98 days), 6, (180–191 days), and 9 months (269–286 days); all infants were born at term (37–41 weeks of gestational age, weight range 2,500–4,500 g) and healthy expect for minor illnesses. Infants were only tested when awake and calm. All were raised in a Caucasian environment where they were unfamiliar with African faces or individuals; 46 infants completed all assessments, 21 additional infants provided data at two adjacent ages, the remainder provided data at one age. Sample sizes vary depending on the analysis.

#### 2.2.2. Ethical guidelines

This research was conducted under the aegis of the University of Bielefeld and followed their guidelines (Grunds$\ddot{\text{a}}$tze zur Sicherung guter wissenschaftlicher Praxis an der Universit$\ddot{\text{a}}$t Bielefeld, authored in 2000); no special ethics review was required. Written consent was obtained from all parents prior to their infant's participation. The study accorded with ethical guidelines of the American Psychological Association and the Society for Research in Child Development.

#### 2.2.3. Presentation, data reduction, stimuli

Prior to each paired presentation was a three second interval, during which a white circle appeared accompanied by a sound. This midline-oriented the infant's gaze, then disappeared before the next trial began. Eye movements were video-recorded then analyzed off-line for gaze direction. Each trial was defined by 130 video frames. With 25 frames per second, each trial was slightly more than 5 s. The stimuli were 24 pairs of frontal view female faces. One stimulus was from set C Caucasian faces, and one from set A African faces. The procedure was constrained so that Caucasian faces appeared equal times on the left and right. Among the six Caucasian and six African faces, each appeared four times randomly paired with a face of the other race. Video frame fixation frequencies for the Caucasian and African faces were summed for each trial. The variable of interest was the difference: Caucasian sum minus the African sum for infant *i* on trial *t* denoted, as noted above, *d*_*it*_. Inter-rater reliabilities for shifts in the eye-movements were in the 0.90 s. Note, importantly, the procedure was a Fantz preference procedure, not an other race effect paradigm. Also, the 5 s trial length was selected because it was thought a longer length trial might lead, with 24 trials, to possible habituation effects which it was desired to avoid.

### 2.3. Developing a model for *i*

To specify the probability distributions associated with long and short responses, graphs of data for all infants on all assessment occasions, were examined, letting the data drive the selection of the probability model in the spirit of van der Laan (2015) who defines statistics as learning from data. Second, a model structure linking these distributions is required which leads to a model for *i*. Finally, a procedure for estimating each *i*'s unknown model parameters is required (see Appendix in Supplementary Materials). The responses of each *i* are viewed as a random sample from *i*'s *own population of responses* which implies there is no stochastic dependency between trials; this issue is revisited much later. Thus, there is no assumed common population of infant responses. Aggregation over infants occurs at the parameter level, the level above the observed data level. Infants have their own parameter values. Standard errors and confidence intervals are available at the individual infant level with the bootstrap (Efron and Tibshirani, 1993).

### 2.4. Specifying probability distributions for *i*

**Figure 1:**
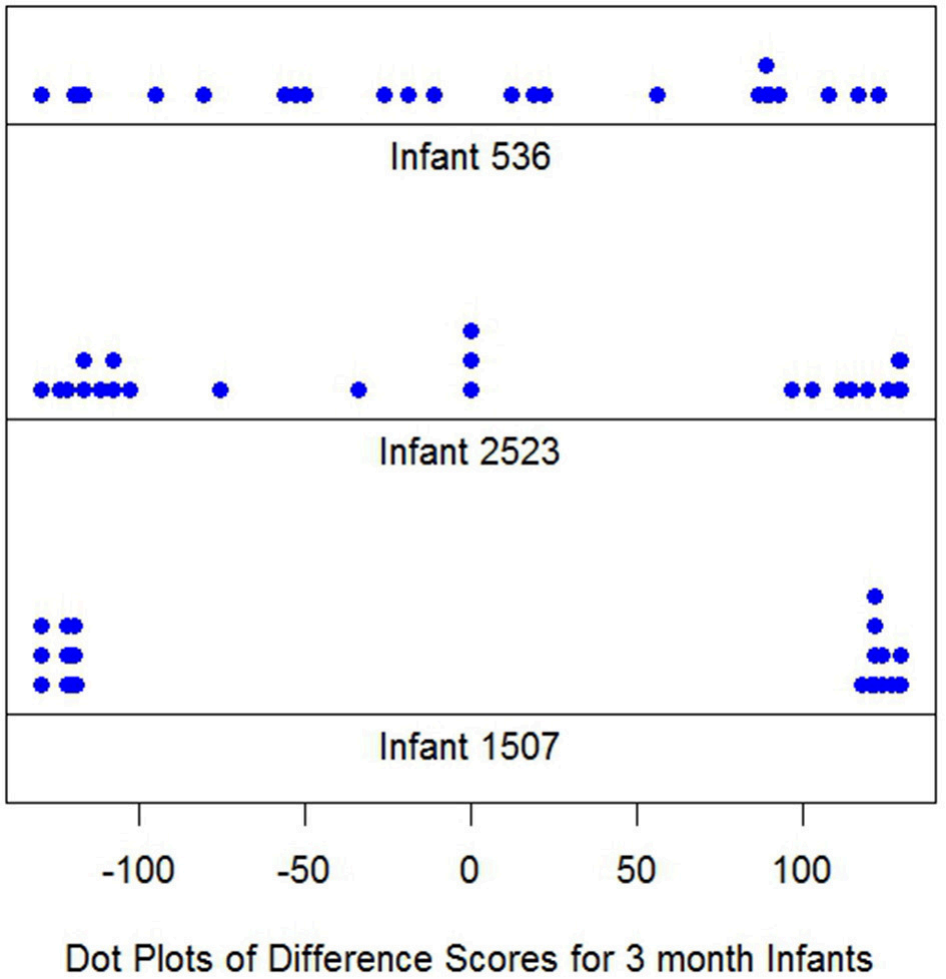
Dotplots *d* for 24 trials for 3 month infants.

Figure 1 displays difference score dot plots for 24 trials for three 3-month infants. Each dot represents one difference score, *d*_*it*_. It is immediately obvious there are large differences among these infants. Infant *i* 536 displays a roughly uniform (rectangularly shaped) distribution of scores over the possible range of difference scores from −130 to 130. However, the responses of *i* 1507, are clustered at the extremes of the response range near −130 or 130.

This infant would appear to be the classic long looker. Judging the distribution of the dots at each extreme, near −130 in one case and near 130 in the other is difficult, but each appears to be approximately uniform in distribution over a narrow interval. In contrast, *i* 2523's dot plot appears to be a mixture of looks resembling both *i* 536 and *i* 1507 or at least is different from either of the other infants. Recall *i* 2523's responses are displayed above.

Note it is the *shape* which distinguishes the dot plot differences among these infants. The means of the *d*_*it*_ differences for all infants are similar. The means are, top to bottom, Figure 1, 1.21, 1.67, and 0.58. The graphs illustrate Cohen's (1972) concern: The averages convey little about how different the looking distributions for these three infants actually are.

The variances in Figure 1 increase: 7390, 11784 and 15974, from top to bottom. Nothing resembling normality is displayed in Figure 1, and the empirical distributions, for each *i*, easily reject normality (Shapiro and Wilk, 1965), as do the distributions of virtually all of the 3 month's infant data and most of response distributions of the 6 and 9 month data as well.

The infants in Figure 1 were selected to illustrate individual differences in response distributions. Not all infant dot plots appeared as orderly as the Figure 1 displays, but following the examination of 187 dot plots Figure 1 is representative of the individual distributions found.

Now to specify the distributions: Short responses for *i* will be modeled by one uniform distribution. Long responses for *i* will be modeled by two disjoint uniform distributions, one each near the boundaries of the range of the response times. Thus, each *i* has three uniform distributions (i.e., three rectangles) all her own: One for short responses, and two for long responses. However, as will be seen, when the algebra of the model is considered, some distributions vanish for some infants.

**Figure 2:**
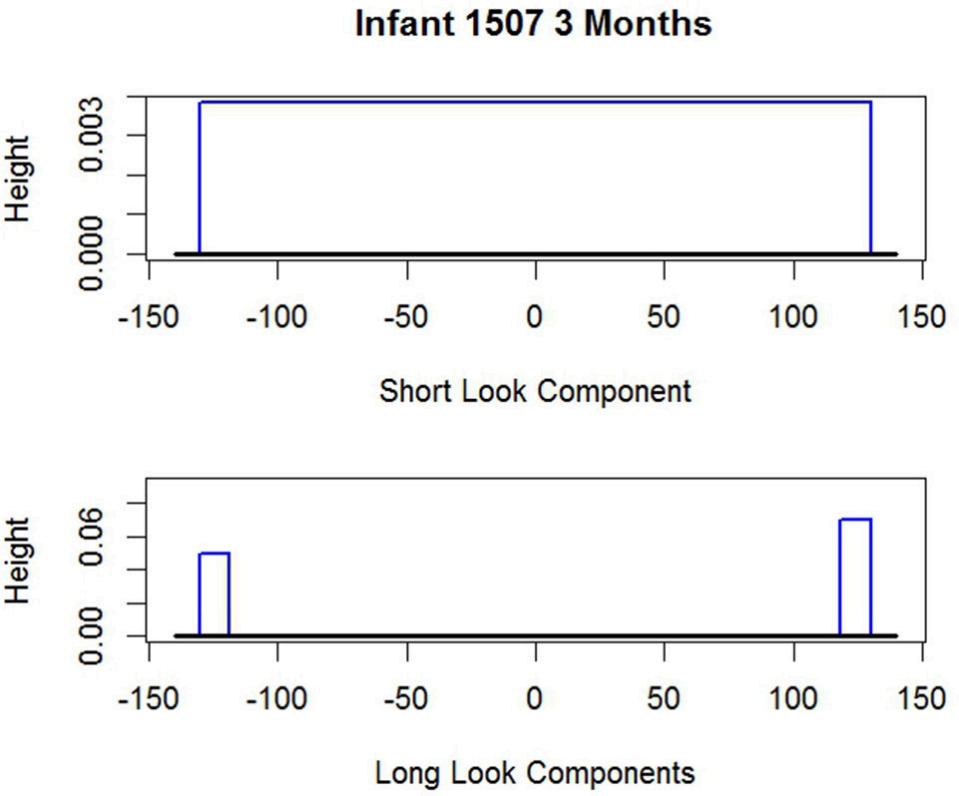
Two estimated probability distributions for *i* 1507. Short look component endpoints: ${â}_{i}=-130,{\hat{f}}_{i}=130$. Long look component endpoints (two components): ${â}_{i}=-130,{\hat{b}}_{i}=-119,{ê}_{i}=118,{\hat{f}}_{i}=130;{\hat{\pi }}_{i}=0,$ the probability *i* is SL, so the Short Look Component vanishes.

**Figure 3:**
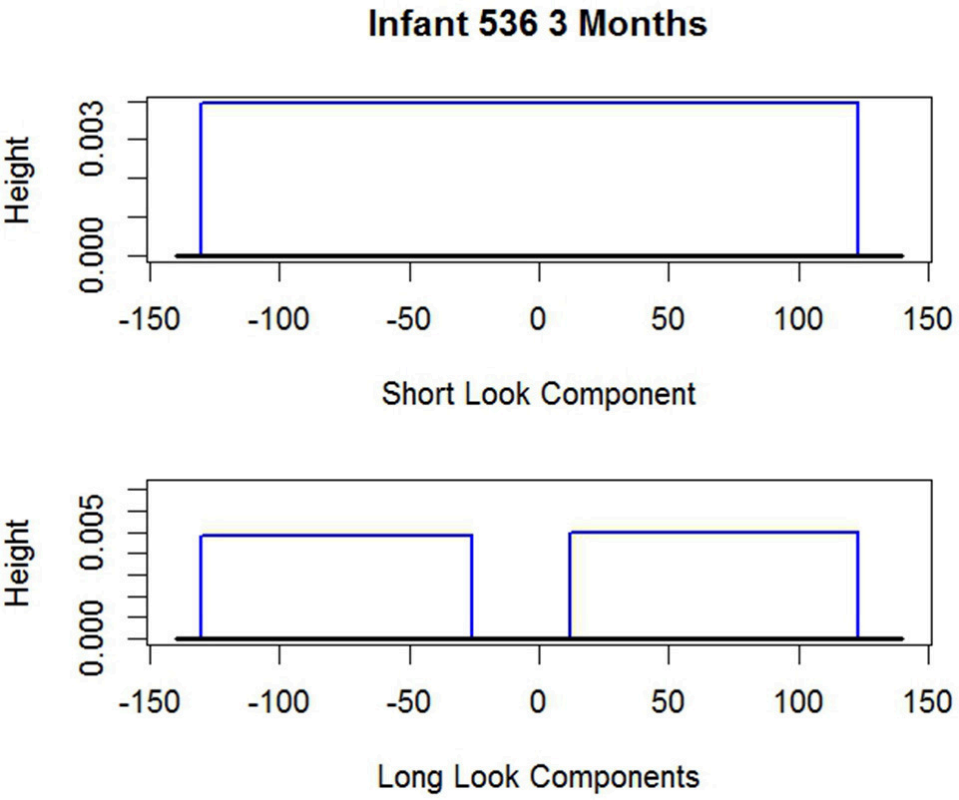
Two estimated probability distributions for *i* 536. Short look component endpoints: ${â}_{i}=-130,{\hat{f}}_{i}=123$. Long look component endpoints (two components): ${â}_{i}=-130,{\hat{b}}_{i}=-26,{ê}_{i}=12,{\hat{f}}_{i}=123;{\hat{\pi }}_{i}=1,$ the probability *i* is SL, so the Long Look Components vanish.

To anticipate, examples of these estimated distributions are given in Figures 2, 3 for *i* 1507 and *i* 536 respectively. The shape of the long and short distributions are the same for all infants. But the endpoints of the uniforms (rectangles) and consequently their heights are unique for each *i*. The parameters noted in the figure captions are defined below.

#### 2.4.1. Linking *i*'s long and short distributions

Each *i* has unique probability distributions for long and short looking. As earlier noted, *i*'s parameter π_*i*_ is the probability of *i* being a short looker and is the proportion of short responses for *i*. 1−π_*i*_ is the proportion of long responses. When it is important to distinguish the assessment occasion or age, the subscript *o*, *o* = 3, 6, 9, π_*io*_ will appear. If all responses for *i* are short, π_*i*_ = 1. If all are long, π_*i*_ = 0. Which *d*_*it*_ are short and which long is determined by a standard an EM algorithm classifier (Everitt and Hand, 1981; Titterington et al., 1985; McLachlan and Basford, 1988; McLachlan and Peel, 2000).

In what follows, *u* denotes a uniform distribution (or additive pieces of uniforms) defined on the real interval, for example *a* to *f*, and denoted *u*(*a, f*), with *u*(*a, f*) = 1/(*f*−*a*), the ordinate height of the uniform distribution. This is just a rectangle with length *f*−*a* and area one.

## 3. The model for *i*

*i*'s model is:

(1) $$\begin{array}{r} \underset{\text{observed}}{\underset{︸}{u_{\text{mix}}\left(d_{it}\right)}}=\underset{\text{latent}}{\underset{︸}{\pi_{i}u_{\text{short}}\left(a_{i},f_{i}\right)+\left(1-\pi_{i}\right)u_{\text{long}}\left(d_{it}\right)}} \end{array}$$

Note that Equation (1) depends on *i* but does not depend on *t*. The subscripts short and long denoting the corresponding short and long distributions, and lettered arguments, *a*_*i*_ and *f*_*i*_ denoting the endpoints of the uniform for *i*. The long distribution *u*_long_(*d*_*it*_) is composed of two weighted uniform distributions *u*_long_(*d*_*it*_) = λ_*i*_*u*(*a*_*i*_, *b*_*i*_)+(1−λ_*i*_)*u*(*e*_*i*_, *f*_*i*_) and *u*_short_(*a*_*i*_, *f*_*i*_) = *u*(*a*_*i*_, *f*_*i*_). In the notation *u*_mix_ and *u*_long_ both taking arguments *d*_*it*_ are mixtures of uniform distributions. The three distinct distributions, one short, and one long itself consisting of two distributions, are the mixture distribution components. The parameters *a*_*i*_ < *b*_*i*_ < 0 < *e*_*i*_ < *f*_*i*_ are component (rectangle) endpoints. The parameters λ_*i*_ denotes the proportion of responses that are negative (*d*_*it*_ < 0) and 1−λ_*i*_ the proportion that are positive (*d*_*it*_ > 0).

As noted, Figures 2, 3 give the long and short components for *i* 1507 and *i* 536 respectively. The figure captions give the estimated endpoints. Neither figure reflects the values of ${\hat{\pi }}_{i}$ which alters the heights of the components. For *i* 1507, Figure 2, ${\hat{\pi }}_{i}=0$, so all *d*_*it*_ are estimated to have come from *i*'s long distribution. Consequently because the ${\hat{\pi }}_{i}$ weight of the short component is zero the short component vanishes. For *i* 536, Figure 3, all responses are estimated to have come from the short component, so ${\hat{\pi }}_{536}=1$ and so the long components vanish. The corresponding figure for *i* 2523 is not given, but his graph is similar; his data appear above. *i* 2523 is a mixed looker, with ${\hat{\pi }}_{i}=0.36$, so most of his responses are estimated to have come from his long distribution.

What has been provided are tailor-made models for an *i*'s individual responses. Eye-tracking methodology typically aggregates over infants and often over their responses as well, sometimes *tens of thousands* of them (Papageorgiou et al., 2014, Figure 1).

Equation (1) is a latent variables model because, as noted above, the knowledge of which distribution, long or short, gave rise to a particular *d*_*it*_ is unknown. Observations are assumed to have come from the mixture distribution or the left side of Equation (1); inference focuses on estimating the parameters on the latent right side. As noted, the central goal is to give each *d*_*it*_ a probabilistic interpretation as to which component distribution, long or short, *d*_*it*_ “came from.” The result are estimates of the form

$$\hat{P}\left(i\text{ gave a short look on trial }t\text{ given }d_{it}\right)$$

The average of these probabilities is ${\hat{\pi }}_{i}$.

## 4. Results

The results are in three parts with subsections. Part one considers the model and data agreement. Part two provides definitions of SL and LL based on ${\hat{\pi }}_{i}$ and considers each infant's trajectory of change from LL to SL. Part three illustrates how the development of looking style impacts same or other race face preferences.

### 4.1. Model and data agreement

**Figure 4:**
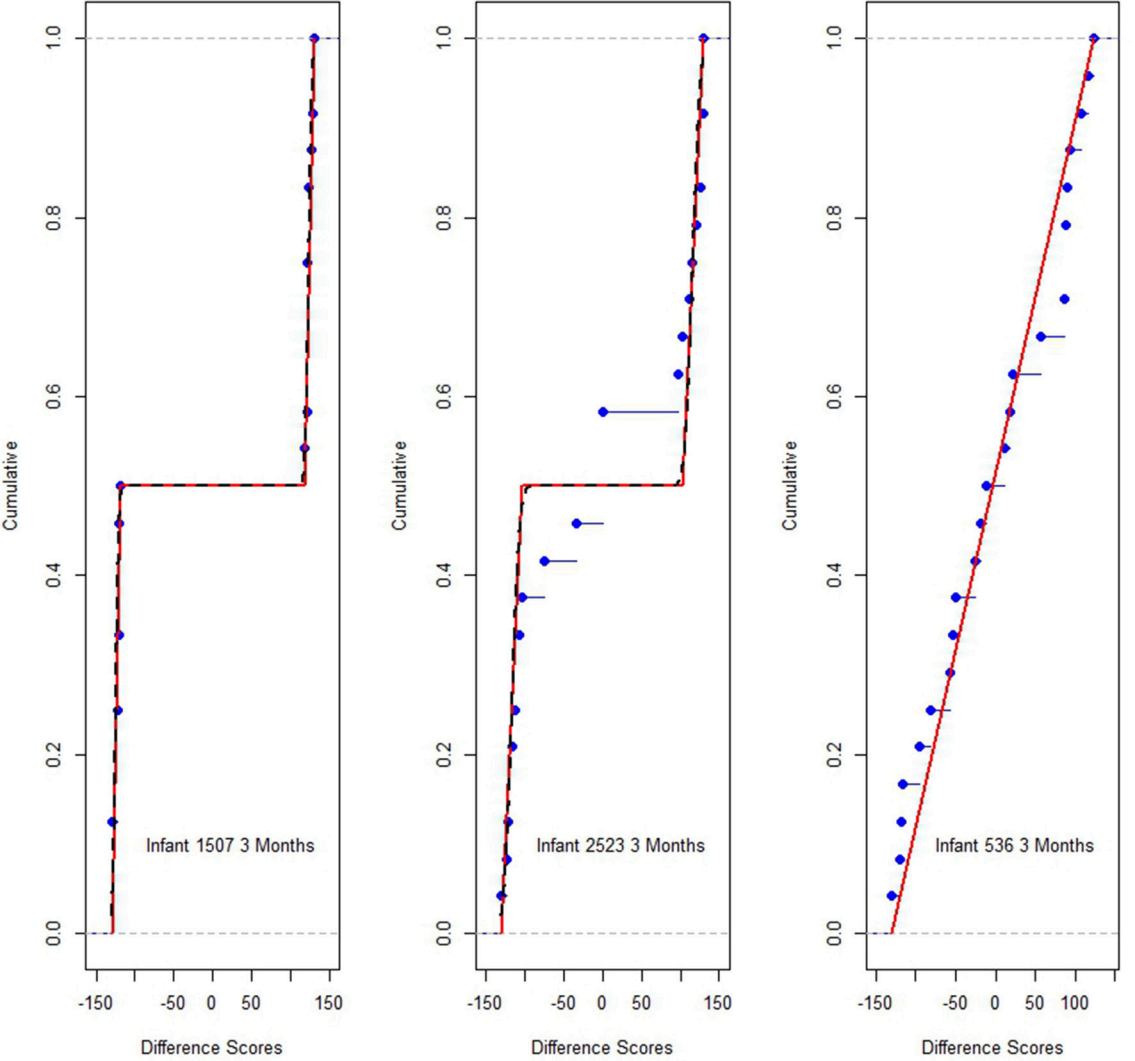
Estimation probability distribution functions (lines), and empirical distribution function responses *d* points, for infants of Figure 1.

Figure 4 shows the correspondence between the model estimates and data for the Figure 1 infants. The points (dots) graph each *i*'s empirical distribution function while the lines graph *i*'s model estimated cumulative distribution functions associated with Equation (1). The points should track the lines, within stochastic variation, if the model is a plausible one. The graphs suggest good agreement between the model and data.

To collectively express the correspondence between model and data for all *i* and for occasions *o* = 3, 6, 9 the estimated variance under the model, and sample variance were compared for each *i* and *o*. The ratios of the average model variance to average sample variance (just the variance of the *d*_*it*_ for *i*) was 0.84, 1.05, 1.15, for *o* = 3, 6 and 9 months respectively. These findings suggests some under fitting at 3 months (there was more variation in the data than the model could account for) and some over fitting at 6 and 9 months (the model was somewhat more complicated than perhaps needed); combining the three ages however, the average ratio is almost exactly one. The models and data appear in generally good agreement.

### 4.2. Two SL definitions

There are two natural criteria for explicitly defining SL and LL based on π_*i*_:

$$i\text{ is SL}\Leftrightarrow \{\begin{array}{ll} \pi_{i}>1/2, & \text{weak SL criterion}\\ \pi_{i}=1, & \text{strong SL criterion} \end{array}$$

and equivalently, *i* is LL. Thus, the theory enables SL and LL to have two versions: weak and strong. In applications, the estimate ${\hat{\pi }}_{i}$ replaces π_*i*_. The weak criterion would be Bayes-rule optimal if the model were fully specified, i.e., parameter values known (Ripley, 1996, p. 19; McLachlan and Peel, 2000, pp. 30–31). Such results do not apply here because parameter estimates replace parameters.

### 4.3. Developmental trajectories in looking style

**Figure 5:**
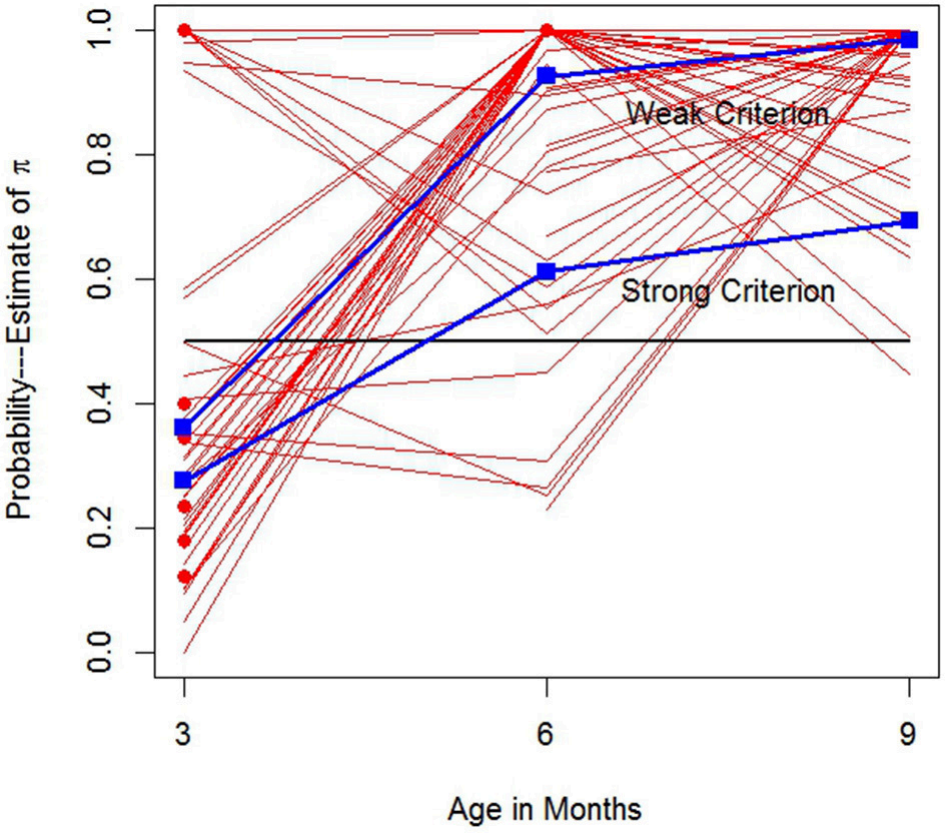
${\hat{\pi }}_{i}$ growth trajectories. Solid lines: Trajectories for infants assessed at two or more ages. Dots: Infants assessed at one age. Rectangles: Means for infants satisfying each looking criterion.

Figure 5 displays the ${\hat{\pi }}_{i}$ and trajectories associated with all 187 observations (reminder: π_*io*_ denotes *i*'s π parameter on occasion *o*). Each solid line is a ${\hat{\pi }}_{i}$ trajectory for an infant *i*; 46 infants were assessed at three ages, 21 others at two ages, and 7 designated with circles, were assessed once. For months 3, 6 and 9, there are 58, 67, and 62 respectively, ${\hat{\pi }}_{i}$. Not all infants are uniquely identified in Figure 5 because many infants shared similar or identical trajectories. For example, five infants displayed strong SL responses with $\hat{\pi }=1$ at all three ages, represented by the horizontal line at 1. While most infants displayed generally increasing values of ${\hat{\pi }}_{i}$ over age, among those 46 infants with data at all three age, more than 15% of them displayed either ${\hat{\pi }}_{i}$ values of 1 at all three ages, or clear discrete just-step-like performance with ${\hat{\pi }}_{i3}=0$ and ${\hat{\pi }}_{i6}={\hat{\pi }}_{i9}=1$. Several other infants were highly similar. Figure 5 reveals striking individual differences in (estimated) trajectory paths.

Trajectories or points below the horizontal line at one-half are weakly LL, while those above, are at least weakly SL. Bronson (1994) suggested that infants become adult-like in their looking behavior by 3 months or so. These data would suggest otherwise. Just 21 of 58 or 36% of the ${\hat{\pi }}_{i3}$ were weakly SL. The square points denote the proportions of infants at all three ages satisfying the weak and strong criteria. Figure 5 clearly shows that by 6 months there is striking improvement in SL and by 9 months nearly all infants display weak SL criteria, presumably similar to adult responding. Yet even at 9 months only 43/62 or 69% display strongly SL. It appears some infants may not fully achieve strong SL until later in development.

### 4.4. Weak SL probability analysis

If the weak criterion is considered, in which case infants with ${\hat{\pi }}_{io}>0.5$ are defined as SL, while those with ${\hat{\pi }}_{io}<0.5$ are LL, the results are well ordered. The estimated conditional probabilities are shown in Table 1 with the notation for example, $\hat{P}\left(\text{SL}6|\text{LL}3\right)$ specifying the conditional probability of being SL at 6 months given having been LL at 3 months. The second row of Table 1 reveals that once weak SL, an infant with near certainty remains SL. However, focussing on the weak criterion can be deceptive as many infants, even at 9 months, have not achieved the level of SL some of their peers achieved at a much earlier age, as already noted.

**Table 1:** Selected SL and LL Conditional Probabilities at 3 and 6, 6 and 9, and 3 and 9 months.

**3 and 6, *n* = 51**	**6 and 9, *n* = 61**	**3 and 9, *n* = 47**
$\hat{P}\left(SL6|LL3\right)=0.87$	$\hat{P}\left(SL9|LL6\right)=1.0$	$\hat{P}\left(SL9|LL3\right)=0.97$
$\hat{P}\left(SL6|SL3\right)=1$	$\hat{P}\left(SL9|SL6\right)=0.98$	$\hat{P}\left(SL9|SL3\right)=1$
$\hat{P}\left(LL6|LL3\right)=0.12$	$\hat{P}\left(LL9|LL6\right)=0$	$\hat{P}\left(LL9|LL3\right)=0.03$

*For example SL3, is shortlooking at 3 months. LL6 is long looking at 6 months*.

### 4.5. Face preference and looking style

Do infants prefer same race or other race faces at ages 3, 6, and 9 months, and how does this preference to change over age? As noted above, this question has been addressed by other investigators (e.g., Liu et al., 2015) but not with longitudinal data and without benefit of knowing each *i*'s looking style.

#### 4.5.1. Face preference unconditioned on looking style

The following employs commonly employed *t*-test procedures (e.g., Sangrigoli and de Schonen, 2004), but ignores the role of ${\hat{\pi }}_{io}$ which is considered in the next section. ${\bar{d}}_{io}=\underset{t}{\sum }d_{ito}/24$ is the mean for *i* on occasion *o*, with ${\bar{d}}_{o}=\underset{i}{\sum }{\bar{d}}_{io}/n_{o}$, the overall mean on *o*, with *n*_*o*_ sample size. Recall negative means indicate other race face preferences, positive means same race face preference. The first row of Table 2, provides results for 3 month infants. The overall mean ${\bar{d}}_{3}=-2.81$, suggests a preference for the African face at 3 months but the *t*-test is insignificant. At 6 and 9 months significant other face preferences were found, as Table 2 column four indicates.

**Table 2:** Difference Score Means ${\bar{d}}_{o}$ Unconditional, and Conditional on SL and LL at three ages.

***o***	***n***	**${\hat{\pi }}_{o}$**	**${\bar{d}}_{o}$**	**${\bar{d}}_{o}|\text{SL}$**	**${\bar{d}}_{o}|\text{LL}$**
3	21.37	0.50(0.05)	−2.81(2.07), *ns*	6.50(4.14), 0.05	−8.11(2.33), 0.001
6	62.5	0.89(0.03)	−4.22(3.37), 0.02	−4.38(1.75), 0.02	−5.00(12.33), *ns*
9	61.1	0.94(0.02)	−6.92(1.52), 0.001	−6.53(1.40), 0.001	−30.67

*o = age; 21, 37 denotes number conditioned on SL $\left({\hat{\pi }}_{i}>0.5\right)$, conditioned on LL $\left({\hat{\pi }}_{i}<0.5\right)$ respectively; n = 21 + 37 sample size. Standard errors are in parenthesis followed by one sample two-tailed t significance level under zero null*.

#### 4.5.2. Face preference conditioned on looking style, I

A very different perspective for 3 month infants is provided by conditioning on looking style. (All tests are two-tailed.) Obtain ${\bar{d}}_{3}|SL=6.50$ by averaging over ${\bar{d}}_{i3}$ for those 21 infants for which ${\hat{\pi }}_{i3}>1/2$; also compute ${\bar{d}}_{3}|LL=-8.11$ for those 37 infants with ${\hat{\pi }}_{i3}<1/2$. Both of these means are significantly different from zero, as Table 2 indicates, and are very different from each other (Welch's *t*_(41.6)_ = 3.73, *p* < 0.0005). SL infants at 3 months prefer their own race faces; LL 3 month infants prefer African faces. Contrasted with the unconditional results, conditioning on looking style provides dramatically different results for the 3 month infants.

At 6 and 9 months, the unconditional means, ${\bar{d}}_{6}=-4.22$ and ${\bar{d}}_{9}=-6.92$ are nearly the same as their corresponding SL conditional means, as Table 2 indicates, because there are very few (weak criterion) LL infants, just 5 at 6 months, (with LL proportion $1-{\overset{-}{\pi }}_{6}=0.11$) and only one LL infant at 9 months (with LL proportion $1-{\overset{-}{\pi }}_{9}=0.06$). The results are essentially unchanged when just those infants assessed at all three ages are examined.

#### 4.5.3. Face preference conditioned on looking style, II

**Figure 6:**
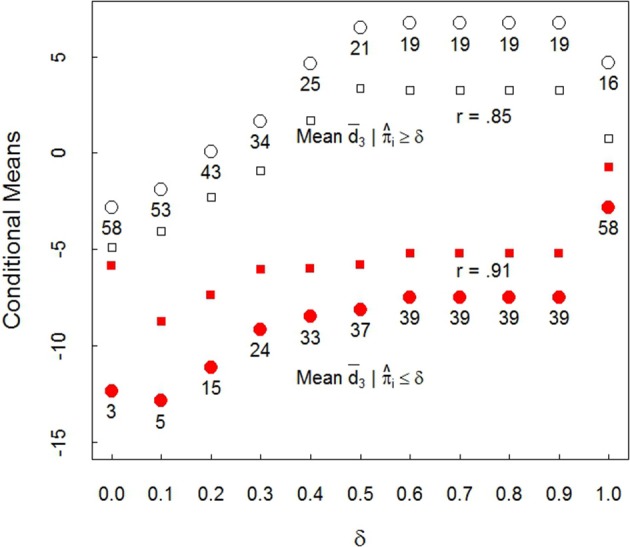
Three month infant plot of means ${\bar{d}}_{3}|\hat{\pi }\leq \delta$, filled circles and ${\bar{d}}_{3}|\hat{\pi }\geq \delta$ unfilled circles, against δ. Filled and open squares are standard errors. *r* is the conditional mean with δ correlation. Numbers display sample size in each mean.

It is instructive to extend the above analysis in the following way: Set δ to take discrete values in the interval from zero to one: δ = 0, 0.1, 0.2, …, 1. For the 3 month infants, using ${\bar{d}}_{i3}$, compute the mean of the ${\bar{d}}_{i3}$ for which the corresponding ${\hat{\pi }}_{i3}\leq \delta$. Thus, the mean ${\bar{d}}_{3}$ is computed over values of ${\bar{d}}_{i3}$ for which *i*'s associated ${\hat{\pi }}_{i3}$ is less than or equal to δ. And similarly for the ${\bar{d}}_{i3}$ for which the corresponding ${\hat{\pi }}_{i3}\geq \delta$. These resulting means are denoted as ${\bar{d}}_{3}|{\hat{\pi }}_{i3}\leq \delta$ and ${\bar{d}}_{3}|{\hat{\pi }}_{i3}\geq \delta$, respectively. The results of this analysis appear in Figure 6. The filled circles are for the averages ${\bar{d}}_{3}|{\hat{\pi }}_{i}\leq \delta$. The unfilled circles are the averages ${\bar{d}}_{3}|{\hat{\pi }}_{i}\geq \delta$. The graph plots these means against the values of δ (only at δ = 0 and δ = 1 were any ${\hat{\pi }}_{i3}$ equal to δ). Note also that unconditional mean, −2.81, is given by the left most unfilled (δ = 0) and the right most filled (δ = 1) circles.

Consider δ = 1/2. The unfilled circle with 21 below it, and the filled circle with 37 below it, are the corresponding first row values 6.50 and −8.11 from Table 2. The unfilled squares and filled squares indicate the sizes of the associated standard errors. The graph makes clear it matters little at which values of δ and associated π on which conditioning occurs, the corresponding differences in conditional means are widely different, and generally several standard errors apart. Clearly conditioning on looking style has a dramatic effect on 3 month infant face preference results.

Figure 6 also reveals that as δ increases, and consequently, ${\hat{\pi }}_{i3}$ increases, the magnitude of the mean ${\bar{d}}_{3}|{\hat{\pi }}_{i3}\leq \delta$ and the mean ${\bar{d}}_{3}|{\hat{\pi }}_{i3}\geq \delta$ increase in the direction of same race face preference for those with ${\hat{\pi }}_{i3}$ up to about 1/2. The function is roughly constant for those values of δ > 1/2 except for δ = 1 because there are few ${\hat{\pi }}_{i3}$ in the broad interval from about 1/2 to nearly 1.

That the conditional means increase as δ increases is predictable: As ${\hat{\pi }}_{i3}$ increases, the probability of SL increases, and the larger the values of ${\hat{\pi }}_{i3}$, the larger the associated conditional mean. This graph reveals that it is not simply that infants with weak SL (with ${\hat{\pi }}_{i3}>1/2$) prefer same race faces, as Table 2 demonstrated. Figure 6 shows that as the magnitude of ${\hat{\pi }}_{i3}$ increases, so does the magnitude of preference for same race faces: Face preference and looking style track each other.

#### 4.5.4. Are looking style and face preference independent?

View the filled and unfilled circles in Figure 6 as two regressions. A test of the independence of looking style and same or other race face preference follows easily. Under independence, as in conventional regression, the regressions have zero slope (see Appendix F in Supplementary Materials). A bootstrap hypothesis test (Efron and Tibshirani, 1993) rejects independence for both regressions (*p* < 0.01) so face preference and looking style are dependent. Graphs constructed for the 6 and 9 month groups were similar. However, there are very few LL infants at these ages, half of the corresponding filled circles at 6 and 9 months are based on from one to nine infants, too few for any firm statements to be made.

Next, consider an alternative perspective by ignoring the magnitude of ${\bar{d}}_{i}$, but tally the relative frequencies while, using the weak SL criterion, and conditioning on looking style. If ${\bar{d}}_{i}<0$ the other race face is preferred (ORP) while if ${\bar{d}}_{i}>0$ the same race is preferred (SRP). Table 3 reports these findings. Consider first the unconditional $\hat{P}\left(\text{ORP}\right)$ in column 3. At all ages $\hat{P}\left(\text{ORP}\right)$ dominates $\hat{P}\left(\text{SRP}\right)$ in column 4, a finding parallel to the results reported in Table 2. However, again the value of conditioning is revealed. Note that $1-\hat{P}\left(\text{ORP}|\text{SL}\right)=\hat{P}\left(\text{SRP}|\text{SL}\right)=0.67$ at 3 months, but $\hat{P}\left(\text{ORP}|\text{SL}\right)=0.64$ and 0.79 at 6 and 9 months. Condition on SL, and the results parallel the findings in Table 2, Column 5.

**Table 3:** Unconditional and conditional probabilities associated with SRP and ORP Face Preferences.

***o***	***n***	**$\hat{P}\left(\text{ORP}\right)$**	**$\hat{P}\left(\text{SRP}\right)$**	***P*_1_**	***P*_2_**	***P*_1_ − *P*_2_**
3	58	0.55(0.06)	0.45(0.06)	0.22(0.07)	0.33(0.10)	−0.11(0.07)
6	67	0.64(0.06)	0.36(0.06)	0.93(0.04)	0.64(0.06)	0.29(0.06)
9	62	0.79(0.05)	0.21(0.06)	0.98(0.04)	0.79(0.07)	0.19(0.05)

*o = age. Bootstrap standard errors are in parentheses. $P_{1}=\hat{P}\left(\text{SL}|\text{ORP}\right),\;P_{2}=\hat{P}\left(\text{ORP}|\text{SL}\right)$*.

#### 4.5.5. Does looking style change precede face preference change?

Table 3 makes it possible to address the following important question: Does SL precede other race preference in development or does it lag behind? It is a straight forward to check the methodological rationale for addressing this question which is introduced with a story: Regard the number line with marks at 3 and 9 as a path, and think of infant *i* traversing this path starting from the left of 3 and traveling on this path from left to right past 3 and toward 9. Define *B* = {*i* passes 3}, and *C* = {*i* passes 9}. Inquiries about *both* events are made as *i* travels: Has *B* occurred? Has *C* occurred? Critically, *C* occurs only if *B* has occurred, so 1 = *P*(*B*|*C*) > *P*(*C*|*B*). The conditioning event *C* in the left hand larger probability, here *P*(*B*|*C*), is the *later* event to occur. In error prone data with *estimated* probabilities, the inequality should hold, but $1=\hat{P}\left(B|C\right)$, would not be expected to occur. Should *i* travel in the reverse direction passing 9 first and then 3, 1 = *P*(*C*|*B*) > *P*(*B*|*C*). The closer the larger probability is to 1, the greater confidence there is to the inference.

If ORP follows SL in development $P_{1}=\hat{P}\left(\text{SL}|\text{ORP}\right)$ should be larger than $P_{2}=\hat{P}\left(\text{ORP}|\text{SL}\right)$; the reverse should hold if SL follows ORP. The three right-hand columns of Table 3 address this issue. Only at 3 months does *P*_2_ exceed *P*_1_. A bootstrap test of the differences, *P*_1_ − *P*_2_, reveals a two-tailed bootstrap significance (Efron and Tibshirani, 1993) was obtained at 6 (*p* < 0.05) and 9 months (*p* < 0.01), but not at 3 months. Finding that *P*_1_ is very large at 6 and is nearly unity at 9 months, strongly suggests that the development of ORP lags well behind SL in development.

## 5. Discussion

Looking style progresses from LL to SL, although there are large individual differences in the rate of progress; some 3-month infants display SL maturity not achieved by other infants at 9 months. Looking style and face preference were shown to be dependent, with SL well preceding changes in face preference and perhaps playing a causal role in the shift from own race to other race face preferences as the infant develops. Whether looking style changes are method (here paired stimulus array) or stimulus (here faces) dependent is unknown.

These results have shown for the first time that *i*'s looking durations on different trials can be probabilistically assessed as to whether they are long or short, within a conventional paired preference looking paradigm which may have been thought not possible: “If one wants to document…what an infant can do on a *single* trial…” (Aslin, 2012, p. 128, original emphasis) eye-tracking methodology is required. Because conventional procedures were employed, as suggested earlier, the possibility of exploring old data for new insights is possible and likely with little effort or cost. While the method here is specific for paired-array settings, the general strategy should be applicable to other settings.

From a *linking hypothesis* perspective (Aslin, 2012) face preference is linked through *i*'s probability distribution reflected in ${\hat{\pi }}_{i}$, the weight associated with *i*'s SL distribution: Incremental changes in $\hat{\pi }$ are associated with incremental changes in mean face preference at 3 months as Figure 6 shows. Looking style assessment is important because it appears to be a central driver in determining which face stimulus an infant prefers at 3 months, and likely during the earlier weeks in life as well. Figure 6 makes clear the magnitudes of the differences conditioned on looking style at 3 months are huge: The conditional mean differences at δ = 1/2 in Figure 6 (also Table 2 row one) are more than five standard errors apart.

To review, 3 month SL infants prefer their same race faces; LL infants prefer other race faces. When aged 6 and 9 months, the SL 3 month infants remain SL and prefer other race faces. The proportions of LL infants at 6 and 9 months dwindles, so that preferences among these remaining LL infants cannot be specified.

Broadly, the findings replicate other reports (Kelly et al., 2005, 2007; Bar-Haim et al., 2006; Liu et al., 2015). By largely replicating earlier findings, it appears that methodological variations and varying definitions of preference may not be critical. For example, Liu et al. (2015) define preference in terms of differences of *ratios* of looking times based on two face pairings; Bar-Haim et al. (2006) employed commonly used looking time differences based on eight face pairs; differences of 24 pairings were used here.

The issue of looking style has apparently not been discussed as a potential variable in the broad literature concerned with looking responses to facial stimuli. This is likely because heretofore, looking style has been assessed only by employing a pretest, so the role of looking style was never regarded as salient. However the present findings make it clear looking style can be a critical variable to consider at least in preferential looking, and likely other settings as well, in the early weeks of life. A looking style viewpoint also allows for certain alternative explanations and raises issues never considered.

As an example, twelve 3-month infants were sampled from each of three different ethnic groups (Bar-Haim et al., 2006). The Israeli-born infants of Ethiopian origin, were presented with a Caucasian and African pair of faces. “Remarkably, [they]…showed no particular preference…(Bar-Haim et al., 2006, p. 162).” Perhaps the small insignificant preference for Caucasian faces was partly because these infants had experience with faces of both races early on. However the unknown proportion of SL infants in their sample surely would be a contributing factor. Their insignificant result parallels the unconditional insignificant findings reported here for 3 month infants. Had looking style been controlled, a different conclusion might have been rendered, as was the case here.

Recognizing the importance of looking style on infant face preferences makes clear the importance of considering the proportion of SL and LL infants when sampling, and sampling's impact on interpreting results. View sampling as a Bernoulli process, with π_*osl*_ denoting the probability of obtaining a weak SL infant at age *o*, and 1−π_*osl*_ the probability of a weak LL infant. To illustrate, assume SL 3 month infants prefer own faces, LL prefer other race faces and take ${\hat{\pi }}_{3sl}=0.36=21/58$ (Table 2). To demonstrate, unconditionally, a significant own face preference would likely require a substantial proportion of the infants sampled be SL; likely 5 or more SL infants among a sample of 12 are needed. The probabilities are 0.45, 0.20, 0.09 of getting one, two, and three such independently drawn samples with at least 5 SL infants. This describes the sampling situation, from the current perspective, of Bar-Haim et al. (2006). The unknown proportion of SL infants actually sampled likely influenced the outcomes. This possibility presents an intriguing challenge: Separating the influence of looking style from early experience.

Liu et al. (2015) reported preferences for same or other race faces in Chinese infants aged 3, 6, and 9 months. Same race faces were preferred at 3 month, other race faces were preferred at 9 months, but no significant preference was shown at 6 months. Other race faces were preferred at 6 months in the present study. There are two alternative explanations for the Liu et al. (2015) 6 month null result. One is that what occurred in the present unconditional insignificant analysis at 3 months, occurred in their analysis at 6 months. The implications are the same as just discussed: Conditioning on looking style might lead to a different result, although this would seem unlikely if ${\hat{\pi }}_{6sl}=0.93=62/67$ (Table 2) is appropriate for their sample because most infants would likely be SL. This leads to a second possibility.

Liu et al. (2015) assessed Chinese infants; the present study assessed Caucasian infants. It is quite possible that π_*osl*_ might differ for the Caucasian and Chinese infant populations. Freedman (1974) reported a myriad of behavior differences among newborns of seven different ethnic groups. Freedman and Freedman (1969) reported that Chinese-American newborns visually habituate more rapidly than European-American newborns and Japanese newborns lag behind Caucasian newborns in visual following responses (Freedman, 1974, p. 165). Consequently, it does not seem implausible to suspect that the proportions of SL infants in different racial or ethnic groups, given a fixed age, might well be importantly different.

The literature concerned with infant responses to own or other race faces views development largely as a consequence of experiential or socio-cultural learning processes (Anzures et al., 2013). The selection of and comparison with infants from different racial or ethnic groups appears to assume the infants of the same age but from different groups differ primarily in their experiences. The results presented here suggest that looking style can be a potential confounding variable, unless the corresponding population proportion of SL infants, π_*osl*_, is near zero or one or has been controlled by, for example, conditioning on SL or LL subpopulation membership, at least early in life. Another way of measuring the impact of looking style is to note that in Figure 6, for every one tenth increase in δ units, there is roughly a 1.5 unit increase in the mean *d*, at least for 0 ≤ δ ≤ 1/2.

Kelly et al. (2005) reported that 3 month infants prefer same race faces, but newborns show no preference. Null findings are problematic, as they acknowledge, especially in light of earlier literature. Fantz et al. (1962) reported that infants could resolve 40 min. of visual arc with 1/8-in. stripes at 10 in. before they were a month old, and that infants can visually discriminate between patterned stimuli within 48 h after birth (Fantz, 1963). Kelly et al. (2005) note it seems likely “…newborns are able to discriminate between faces …but no group elicits a greater attraction” (Kelly et al., 2005, p. F34). One would expect some infants in the early days of life would display SL, and consequently such SL newborns might display consistent face preferences. What might be the proportion of weak SL newborns?

Beasley (1933, p. 118, Table 3, column 1) reports the proportions of his most mature white newborn visual pursuit responses among his four categorical types of pursuit: 0.21, 0.28, and 0.39, proportions at ages 1 day, 2 to 5 days, and 6 to 12 days respectively. It might be reasonable to suppose that these proportions serve as a surrogate for the proportion of weak SL newborns.

A second source for a newborn SL estimate is provided by regressing for each of the 46 infants with assessment at all three ages, their ${\hat{\pi }}_{i3},{\hat{\pi }}_{i6},{\hat{\pi }}_{i9}$ against occasions *o* = 3, 6, 9 (with intercept estimate constrained to the interval [0,1]) then predicting *i*'s value at 1 week of age, taken as the newborn age, and denoted ${\tilde{\pi }}_{i0.25}.$ The mean of the ${\tilde{\pi }}_{i0.25}$ is 0.32 with the estimated SL proportion ${\hat{\pi }}_{0.25sl}=\underset{i}{\sum }I\left({\tilde{\pi }}_{i0.25}>0.5\right)/46=0.28.$ These two estimates would imply that for Caucasian infants the proportion of SL newborns a week old π_0.25_ *_sl_*, is around 0.25 to 0.30.

More importantly however, because π_.25_ *_sl_* appears to be well away from zero, the actual number of SL newborns in a sample is likely to be non-negligible. Taking ${\hat{\pi }}_{0.25sl}=0.25$, perhaps the expected number of roughly 12 (= 48 ${\hat{\pi }}_{i.25}$ SL newborns in the Kelly et al. (2005) sample of 48 (their same vs. other race face conditions) would show consistent preferences if SL infants could have have been identified. Kelly et al. (2005) interpret the preference shown at 3 months but not at birth as due to learning occurring during the first 3 months. Such a learning explanation is difficult to falsify. An easily falsifiable explanation is that failure to find a face preference at birth or in the earliest days of life is because of the unrecognized importance of looking style differences.

A critical assumption underlies the above framework, as noted earlier, is that the difference scores of each infant are regarded as a random sample from *i*'s population of responses which means that the difference scores are independent and identically distributed random variables within each *i*. Otherwise said, the responses of *i* are a random sample from *i*'s own probability distribution, *u*_mix_(*d*_*it*_) in Equation (1). This assumption cannot be exactly correct, but it does not appear to be importantly wrong. It implies that the order in which the observations were made is irrelevant. This hypothesis can be tested. If the correlation between the pairs (*d*_*it*_, *t*), *t* = 1, 2, 3, …, 24 within each *i* and at each *o* departs from zero, evidence would be provided that the within *i* random sampling assumption is wrong. Bootstrap testing one to three infants were significant, *p* < 0.05, at each *o* = 3, 6, 9 months with samples sizes 58, 67, and 62, respectively. Thus, there is no evidence, within individual infants, of the difference scores being correlated with the trial number. One reason for this lack of correlation may be the following fact. If (random variables) *A* (African) and *T* (trials) have ρ correlation, and *C* (Caucasian) and *T* also have ρ correlation, *C*−*A* = *D*, then *D* and *T* are zero correlated. Basing the analysis on difference scores might have mitigated certain expected trends in data.

Any implementation of the strategy proposed above requires within individual repeated measures. In the paired-stimulus setting Aslin and Fiser (2005) suggest 12 trials are typically obtained; here 24 were obtained. How many replications are needed likely depends on the task, age of infant, the observed variable, and requires study. However, a preliminary analysis suggests that if the first 12 of the 24 responses were employed, results would be little changed from those reported above. Selecting uniform probability distributions as models of infant looking responses is unique. An examination of the data drove this decision. In the absence of a rich theory some consequence of which might specify a particular family of distributions for infant responses, one simply cannot do better than to be guided by the each infant's data. Indeed, the empirical distribution function of *i*'s own data is in fact the non-parametric maximum likelihood estimate of that infant's unknown distribution function, given certain assumptions (Owen, 2001, p. 7–8).

Individual differences remain one of the most challenging and important problems in psychology. How such differences are viewed and conceptualized, if indeed there is any interest in doing so, varies. The most common approach is to partition, at one or more places in the assumed common response distribution over individuals, then label the corresponding groups constructed as different. As noted at the outset, this is how Colombo has defined SL and LL infants (e.g., Colombo et al., 1988). This is not the approach here. Figure 1 shows the *qualitative shape differences* which define individual differences. Formally, infants *i* and *j* are different if and only if the distribution of *i*'s responses is different from the distribution of *j*'s responses. Figure 1 is, for the approach here, a call for modeling each *i*'s response distribution; all *i* share the same *family* of response distributions, additive π-weighted uniform distributions, as in Equation (1).

This research has shown how within-infant looking style differences can inform more substantive issues without additional data collection or technical apparati. Statistical modeling alone is required. But the critical consideration in this modeling, to stress yet again, is modeling *i*'s response distribution. Race–face preferences and looking style were shown to be dependent (Figure 6), with changes in looking style well preceding changes in race–face preference. One consequence: Without considering looking style, inferences concerning race–face preferences can be misleading at least for young infants. From a general substantive perspective, this research leads to the suggestion that looking style may be crucial for understanding a wide variety of infant behaviors never imagined to be impacted by looking style and well beyond the reach of conventional between infant looking style assessment methods.

## Author contributions

IF designed the study, coded the data, and prepared data files. HT did the analysis, modeling and manuscript writing.

### Conflict of interest statement

The authors declare that the research was conducted in the absence of any commercial or financial relationships that could be construed as a potential conflict of interest.
